# Rhamnolipids production from sucrose by engineered *Saccharomyces cerevisiae*

**DOI:** 10.1038/s41598-018-21230-2

**Published:** 2018-02-13

**Authors:** Frederico Mendonça Bahia, Gabriela Carneiro de Almeida, Lorena Pereira de Andrade, Christiane Gonçalves Campos, Lúcio Rezende Queiroz, Rayane Luzia Vieira da Silva, Patrícia Verardi Abdelnur, José Raimundo Corrêa, Maurizio Bettiga, Nádia Skorupa Parachin

**Affiliations:** 1 Department of Molecular Biology, Biological Sciences Institute, University of Brasília (UnB), Campus Darcy Ribeiro, Block K. Postal code: 70.790-900 Brasilia, Federal District, Brazil; 2 Catholic University of Brasília (UCB), Advanced Campus Asa Norte, SGAN 916 Block B Avenue W5, Postal code: 70.790-160 Brasilia, Federal District, Brazil; 3 Brazilian Agricultural Research Corporation, Embrapa Agroenergy, W3 Norte, PqEB, Postal code: 70770-901 Brasília, Federal District, Brazil; 4 Institute of Chemistry, Federal University of Goiás, Campus Samambaia, Postal code: 74690-900 Goiânia, Goiás Brazil; 5 Department of Biology and Biological Engineering, Division of Industrial Biotechnology, Chalmers University of Technology, SE-41296 Gothenburg, Sweden; 6 EviKrets Biobased Processes Consultants, Gibraltarsgatan 40, 41280 Gothenburg, Sweden

## Abstract

Biosurfactants are biological tensioactive agents that can be used in the cosmetic and food industries. Rhamnolipids are glycolipid biosurfactants naturally produced by *Pseudomonas aeruginosa* and are composed of one or two rhamnose molecules linked to beta-hydroxy fatty acid chains. These compounds are green alternatives to petrochemical surfactants, but their large-scale production is still in its infancy, hindered due to pathogenicity of natural producer, high substrate and purification costs and low yields and productivities. This study, for the first time, aimed at producing mono-rhamnolipids from sucrose by recombinant GRAS *Saccharomyces cerevisiae* strains. Six enzymes from *P. aeruginosa* involved in mono-rhamnolipid biosynthesis were functionally expressed in the yeast. Furthermore, its SUC2 invertase gene was disrupted and a sucrose phosphorylase gene from *Pelomonas saccharophila* was also expressed to reduce the pathway’s overall energy requirement. Two strains were constructed aiming to produce mono-rhamnolipids and the pathway’s intermediate dTDP-L-rhamnose. Production of both molecules was analyzed by confocal microscopy and mass spectrometry, respectively. These strains displayed, for the first time as a proof of concept, the potential of production of these molecules by a GRAS eukaryotic microorganism from an inexpensive substrate. These constructs show the potential to further improve rhamnolipids production in a yeast-based industrial bioprocess.

## Introduction

Surfactants - surface active agents - are amphiphilic molecules, which tend to accumulate at the interface between polar and non-polar solvents. Hence, these molecules possess the ability to reduce interfacial and surface tension, leading to enhanced mixing and interaction between dissimilar phases^1^. Consequently, surfactants have a broad range of industrial utilizations, e.g., production and processing of foods, agrochemicals, pharmaceuticals, petroleum, mineral ores, personal care and laundry products, fuel additives, lubricants and many others^2^. This wide spectrum of application translates to an expanding global market that is expected to generate revenues of more than 42 billion USD by 2020, with a 5.5% growth rate p.a.^3^.

Surfactants can be classified into two main groups based on their production process: synthetic surfactants and biosurfactants. The former are produced by organic chemical reactions and are mainly petroleum derived, while the latter are biologically synthesized by microorganisms such as bacteria, fungi and yeast^4^. The use of synthetic surfactants has strong environmental impacts due to high toxicity and low bio-degradability, besides being produced from non-renewable resources. Therefore, advances in biotechnology and increased environment conservation concerns suggest that biosurfactants are promising alternatives to market available surfactants^5^. Their advantages when compared to their petroleum-derived counterparts are improved biodegradability, low toxicity and low irritancy when exposed to human skin^6^. Rhamnolipids (RLs) are the most intensively studied biosurfactants. In addition, RLs are currently approved for use in food products, cosmetics and pharmaceuticals by the US Environmental Protection Agency^1^.

**Figure 1:**
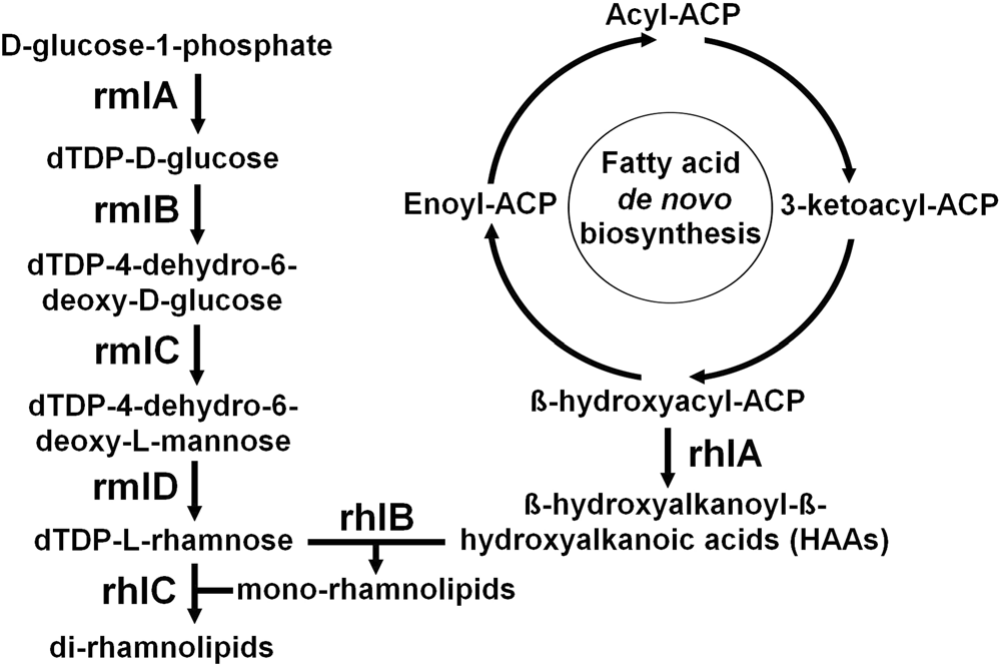
Metabolic pathway for rhamnolipids biosynthesis in *P. aeruginosa*.

Rhamnolipids are produced mainly by the pathogenic gram-negative bacterium *Pseudomonas aeruginosa*. They are composed of one (mono-RLs) or two (di-RLs) rhamnose moieties linked to beta-hydroxy fatty acid chains that vary in number (1 to 3 chains), length (8 to 16 carbons) and degree of unsaturation^7^. RLs production by *P. aeruginosa* involves the coupling of dTDP-L-rhamnose (dTDP-Rha) to a beta-hydroxyalkanoyl-beta-hydroxyalkanoic acid (HAA) or a previously synthesized mono-RL by the rhamnosyltransferases RhlB and RhlC, respectively. The enzyme RhlA converts beta-hydroxyacyl-ACP intermediates from *de novo* fatty acid biosynthesis into HAAs. The conversion of central metabolite D-glucose-1-phosphate (gluc-1-P) to dTDP-Rha is catalyzed by the enzymes RmlA (Glucose-1-phosphate thymidylyltransferase), RmlB (dTDP-glucose 4,6-dehydratase), RmlC (dTDP-4-dehydrorhamnose 3,5-epimerase) and RmlD (dTDP-4-dehydrorhamnose reductase)^5^ (Fig. 1). *P. aeruginosa* has already been extensively studied and modified in order to improve RLs production. Nevertheless, the complex regulation of RLs biosynthesis in this microorganism poses a major challenge when scaling up production. To elucidate the molecular and metabolic mechanisms involved in RLs production, Schmidberger and coworkers^8^ monitored gene expression during batch cultivation of *P. aeruginosa* strain PAO1. It was shown that expression of RhlAB operon is tightly controlled during fermentation by *quorum sensing* mechanisms and co-expressed with virulence factors. Therefore, heterologous expression of *P. aeruginosa* genes involved in RLs biosynthesis in an alternative host would circumvent the regulatory network and the pathogenicity of natural producer. This may allow a more efficient production and reduced costs associated with downstream processes, thereby enhancing the economic feasibility of RLs production.

Some microorganisms were successfully engineered for RLs production, e.g. *Escherichia coli* as well as other *Pseudomonas* species^9^. Up to now, to our knowledge, only bacterial species have been investigated as hosts for RLs production and strategies have recently been compiled^10^. Yields for recombinantly produced RLs reach up to 14.9 g/L by *P. putida* KT2440 using fed-batch fermentation. This value is however many times lower than the ones obtained in *P. aeruginosa* cultures’ supernatants, which can reach up to 120 g/L^10^. Furthermore, several studies on RLs production were conducted using different substrates as carbon source, e.g. diesel, glucose, sunflower oil, oleic acid, sodium dodecyl sulphate, orange fruit peelings, among others^8,9,11–15^. However, current strategies of microbial fermentation for RLs production is only economically feasible when they are required in the composition of high-priced products^10^.

The yeast *Saccharomyces cerevisiae* has been a model organism for decades due to several favorable traits, e.g. its ease cultivation, *GRAS* status and the most advanced genetic tools available for any eukaryotic organism^16^. Indeed, nowadays there are many examples of commercially established large scale production of fine chemicals with *S. cerevisiae* as biocatalyst, examples include the anti-malaria drug precursor amorphadiene (Amyris), vanillin (Evolva), antioxidant resveratrol (Evolva), L-lactic acid (NatureWorks), polyethylene (Braskem) and succinic acid (Reverdia)^17^. Furthermore, the utilization of low cost substrates (e.g. industrial residues and renewable substrates) are currently considered prerequisites for the establishment of an economically feasible bioprocess^5^. In this regard, biomasses from renewable resources are economically attractive alternatives for the production of biofuels and fine chemicals. The use of sucrose as raw material for chemical industries has raised the interest in the past years. Comparatively, the low cost, high availability and purity makes sucrose extremely advantageous for new industrial processes^18^. Indeed, the United States Department of Agriculture (USDA) pointed out that Brazil was the major sucrose producer and exporter in 2015. It adds that sucrose production worldwide is higher than its consumption, making it a low-cost substrate.

Thus, despite having been discovered seventy years ago, there was still no large-scale production of RLs for commercialization up to 2016 due to technological hurdles, namely pathogenicity of natural producer, high raw-material and processing costs and low yield and productivity^5^. Evonik Industries (Essen, DE) is reportedly the first company to produce rhamnolipids in industrial scale, which was announced in a press release from June, 2016^19^. Evonik was granted a patent in April 2017 regarding the production of rhamnolipids from butane by recombinant *Pseudomonas putida* strain^20^. Nevertheless, despite the advances in technology, there is still the need to further decrease production costs to achieve a competitive cost-effective process. In this regard, this study aims to overcome the presented technical challenges by offering an alternative strategy, proving the concept that producing mono-RLs from sucrose with genetically engineered GRAS yeast strains is possible. In this study, two yeast strains were constructed, of which one was transformed with a heterologous pathway for production of dTDP-Rha (RHP strain) and the other with the complete pathway for mono-RLs production (RLP strain). The *de novo* fatty acid biosynthesis cycle that provides the HAA substrate for the RhlA enzyme is already present in the yeast. The production of both molecules of interest by recombinant strains was analyzed by mass spectrometry and confocal microscopy, respectively. The data demonstrated, for the first time as a proof of concept, the potential of a yeast based heterologous RLs and rhamnose production.

## Methods

### Genes and plasmids

**Figure 2:**
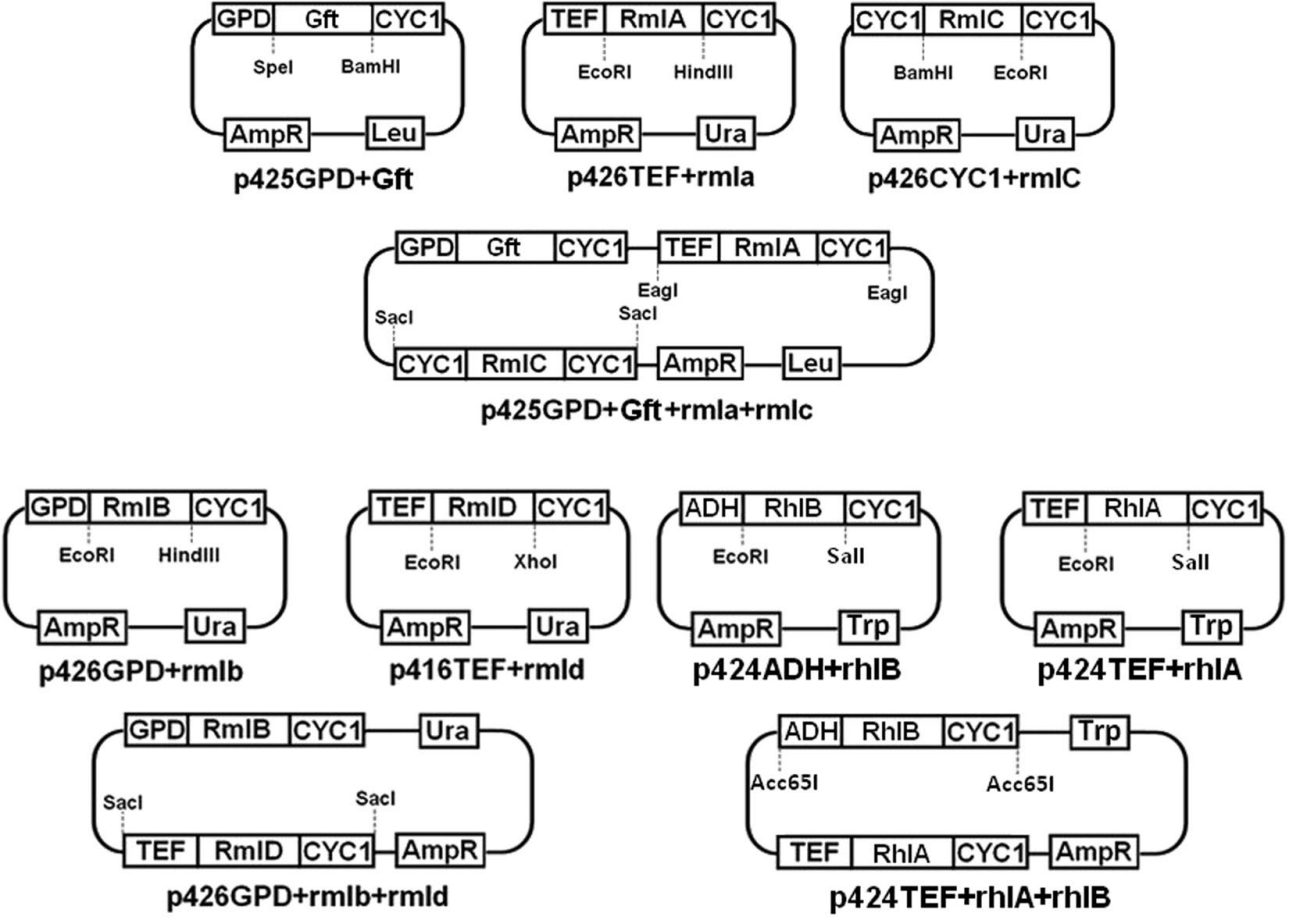
Cloning strategy for plasmids used to transform *S. cerevisiae*.

Seven genes were transferred into the yeast: sucrose phosphorylase gene Gft from *Pelomonas saccharophila* and rmlA, rmlB, rmlC, rmlD, rhlA and rhlB genes from *Pseudomonas aeruginosa* (Fig. 1). The genes were optimized for expression in *S. cerevisiae* and synthesized by the company GenOne and delivered in pBSK series plasmids^21^. Episomal plasmids from Mumberg’s collection^22^ were used as backbone and manipulated via conventional restriction enzyme-mediated cloning methods. Genes’, constructed cassettes’ and plasmids’ sizes can be seen in Table 1. Based on the nucleotide sequences of the target genes and expression cassettes, primer sets were designed and used to amplify DNA fragments by PCR for cloning and confirmation purposes (Supplementary Table S1). Plasmids constructions were confirmed by restriction analysis, PCR and/or sequencing, and the complete cloning strategy can be seen in Fig. 2.

**Table 1:** Genes’, cassettes’ and plasmids’ sizes.

Name	Size (bp)	Name	Size (bp)
Gft	1505	Cassette GPDp-Gft-CYC1t	2503
rmlA	893	Cassette TEFp-rmlA-CYC1t	1604
rmlB	1070	Cassette GPDp-rmlB-CYC1t	2068
rmlC	557	Cassette CYC1p-rmlC-CYC1t	1152
rmlD	921	Cassette TEFp-rmlD-CYC1t	1620
rhlA	899	Cassette TEFp-rhlA-CYC1t	1607
rhlB	1292	Cassette ADHp-rhlB-CYC1t	3037
pBSK	2958	P416TEF	5526
P424ADH	7309	P426CYC1	5411
P424TEF	6246	P426GPD	6637
P425GPD	7760	P426TEF	6352

### *SUC2* gene disruption

*SUC2* gene disruption in *S. cerevisiae* was accomplished by transforming the yeast strains with a KanMX disruption cassette harboring G418 resistance selection marker, as described previously^23^. Two disruption primers were designed to add *SUC2* homologous regions at KanMX’s extremities after amplification using plasmid pUG6 as template^23^. Then, the cassette was transformed into the yeast following previously described protocol^24^. Recombinant colonies were selected in YPD plates containing geneticin (20 g/L peptone, 10 g/L yeast extract, 20 g/L agar and 5 g/L geneticin). Three verification primers were designed in order to align upstream and downstream from the KanMX recombination site and also in the middle of KanMX. These primers were used for verification of *SUC2* disruption in geneticin resistant colonies by colony PCR. Information about the primers described above and the disruption strategy can be seen in Supplementary Fig. S2.

### Strains

#### Bacteria

*E. coli* DH5-α was used for propagation and storage of the recombinant DNA. Bacteria transformation was made following electroporation protocol described in Bio-Rad Laboratories’ electroporator manual. Transformants were grown at 37 °C in Luria-Bertani (LB) medium (5 g/L yeast extract, 10 g/L NaCl and 10 g/L tryptone) with 0.1 g/L ampicillin and confirmed by plasmid extraction protocol followed by restriction analysis and PCR. Positive colonies were stored at −80 °C. See Supplementary Table S3 for information on bacterial strains constructed in this study.

#### Yeast

*S. cerevisiae* strains CEN-PK 102-3A and CEN-PK 113-6B were used as host strains for DNA transformation and expression of recombinant genes. Yeast transformation was made following improved lithium acetate protocol previously described^24^. Transformants were grown at 30 °C in Yeast Nitrogen Base (YNB) medium without amino acids complemented with transformants’ auxotrophic requirements (if any) and glucose or sucrose (20 g/L) as carbon source. Transformants were confirmed by colony PCR and stored at −80 °C. All strains are deposited and catalogued in the Industrial Biotechnology strain repository at Chalmers University of Technology. Relevant information about the yeast strains constructed in this study can be visualized in Table 2.

**Table 2:** Names, relevant genotypes and parental strains of all *S. cerevisiae* strains constructed in this study.

IndBio ID	Name	Relevant genotype	Parental strain
—	**CEN.PK 102-3A**	MATa SUC2 MAL2-8c LEU2 URA3-52	—
SCE IB.0195	**RH1**	MATa SUC2 MAL2-8c LEU2 URA3-52 suc2::KanMX	CEN.PK 102–3 A
SCE IB.0193	**RH2**	MATa SUC2 MAL2-8c LEU2 URA3-52 suc2::KanMX p425GPD	RH1
SCE IB.0197	**RH3 (RHR)**	MATa SUC2 MAL2-8c LEU2 URA3-52 suc2::KanMX p425GPD p426GPD	RH2
SCE IB.0194	**RH4**	MATa SUC2 MAL2–8c LEU2 URA3–52 suc2::KanMX p425GPD Gft rmlA rmlC	RH1
SCE IB.0198	**RH5 (RHP)**	MATa SUC2 MAL2-8c LEU2 URA3-52 suc2::KanMX p425GPD p426GPD GFT RMLA RMLC RMLB RMLD	RH4
—	**CEN.PK 113–6B**	MATα SUC2 MAL2-8c LEU2 URA3-52 TRP1	—
SCE IB.0199	**RL1**	MATα SUC2 MAL2–8c LEU2 URA3–52 TRP1 suc::KanMX	CEN.PK 113–6B
SCE IB.0200	**RL2**	MATα SUC2 MAL2-8c LEU2 URA3-52 TRP1 suc::KanMX p426GPD	RL1
SCE IB.0202	**RL3**	MATα SUC2 MAL2-8c LEU2 URA3-52 TRP1 suc::KanMX p426GPD p425GPD	RL2
SCE IB.0204	**RL4 (RLR)**	MATα SUC2 MAL2-8c LEU2 URA3-52 TRP1 suc::KanMX p426GPD p425GPD p424TEF	RL3
SCE IB.0201	**RL5**	MATα SUC2 MAL2-8c LEU2 URA3-52 TRP1 suc::KanMX p426GPD rmlB rmlD	RL1
SCE IB.0203	**RL6**	MATα SUC2 MAL2-8c LEU2 URA3-52 TRP1 suc::KanMX p426GPD p425GPD rmlB rmlD Gft rmlA rmlC	RL5
SCE IB.0205	**RL7 (RLP)**	MATα SUC2 MAL2-8c LEU2 URA3-52 TRP1 suc::KanMX p426GPD p425GPD p424TEF rmlB rmlD Gft rmlA rmlC rhlA rhlB	RL6

#### Growth rate determination

Recombinant yeast strains were grown in triplicate for 24 hours at 30 °C and 200 rpm. Samples were taken every two hours and OD_600nm_ readings were recorded. The natural logarithm of OD_600nm_ values were plotted over time. The interval where data showed best R-squared for linear regression was chosen to represent the exponential growth phase. The maxim growth rate (μ_Max_) of each strain was determined as the average of the slopes of each triplicate’s linear regression tendency lines.

### Enzymatic assays

#### Total protein extraction

ThermoScientific’s Yeast Protein Extraction Reagent (Y-PER^TM^) was used for total protein extraction as specified by manufacturer’s manual. Extraction samples were immediately used for enzymatic activities assays.

#### RmlA

Coupled colorimetric assay for RmlA activity detection was performed with cellular extract as previously described^25^. The enzymatic reaction contained 50 mM Tris (pH 7.5), 1 mM dithiothreitol, 10% glycerol, 5 mM MgCl_2_, 0.2 mM dTTP, 1 mM D-glucose-1-phosphate and 0.04U pyrophosphatase. One unity (U) of pyrophosphatase is defined as the amount of enzyme required for production of 1μmol P_i_ per minute at pH 7.2 and 25 °C. Reaction was initiated by addition of cellular extract. After incubation at 37 °C for 5 minutes, reaction was terminated by addition of solution containing 0.03% (w/v) green malachite reagent, 0.2% (w/v) ammonium molybdate and 0.05% (v/v) Triton X-100 in 0.7 N HCl and incubation for 5 more minutes. Samples optical densities were measured at 630 nm. Assays were performed in biological triplicates.

### dTDP-L-rhamnose detection

#### Strains

*S. cerevisiae* strain CEN-PK 102-3A suc2::KanMX p425GPD p426GPD Gft rmlA rmlB rmlC rmlD, namely Rhamnose Producing strain (RHP), was tested for dTDP-Rha production. *S. cerevisiae* strain CEN-PK 102-3A suc2::KanMX p425GPD p426GPD, namely Rhamnose Reference strain (RHR), was used as negative control. Strains were grown in YNB medium without aminoacids and sucrose 20 g/L.

#### Extraction

The extraction step followed the protocol adapted from the Gonzalez and Franc methodology^26^. A buffer solution of 75% ethanol (v/v), (10 mM ammonium acetate, pH 7.4) at 85 °C was added to the samples in the ratio 1:1 (v/v) (buffer: sample) and then homogenized in a vortex. Then, samples were incubated for 3 minutes at 85 °C and 800 rpm. Cells were then cooled at −40 °C in thermostatic bath and centrifuged at 5000 rpm and −9 °C for 3 minutes. Cell debris were discarded and supernatants were transferred to new 2.0 mL tubes. Finally, samples were vacuum concentrated and stored at −80 °C.

#### UPLC-MS/MS

The analysis was performed using an ACQUITY UPLC system (Waters, Milford, USA) coupled to a triple quadrupole mass spectrometer (Xevo TQD, Waters) equipped with an electrospray ionization source (ESI-MS). Direct Infusion Mass Spectrometry (DIMS) was used to optimize the analysis conditions by Multiple Reaction Monitoring (MRM). A solution of rhamnose standard at 20 µg/mL was infused. The cone voltage which provided the most intense ion precursor was 40 V. For MS/MS experiment, the collision energy was 20 V. The MS was operated in negative ionization mode, ESI(−)-MS. Instrumental parameters of MS were set as follows: capillary voltage 3500 V, solvation temperature: 450 °C, source temperature: 130 °C, cone gas flow: 20 L/h and solvation gas flow: 700 L/h. The chromatographic separation was achieved using a HSS-T3 reversed phase column with dimensions 2.1 mm × 150 mm × 1.8 µm (Waters). The mobile phase consisted of an aqueous solution of 0.1% formic acid in isocratic elution mode with flow rate of 0.4 mL/min. Column temperature was maintained at 45 °C. The time of each analysis was 5 minutes.

### Rhamnolipids detection

#### Strains

*S. cerevisiae* strain CEN-PK 113–6B suc2::KanMX p425GPD p426GPD p424TEF Gft rmlA rmlB rmlC rmlD rhlA rhlB, namely Rhamnolipid Producing strain (RLP), was tested for mono-RLs production. *S. cerevisiae* strain CEN-PK 113–6B suc2::KanMX p425GPD p426GPD p424TEF, namely Rhamnolipid Reference strain (RLR), was used as negative control. Strains were grown in YNB medium without aminoacids and sucrose 20 g/L.

#### Fluorescence assay

RLP and RLR samples (106 cells) were collected by centrifugation at 3000 g, washed three times in phosphate-buffered saline (PBS) pH 7.4, resuspended in 50 µL of lyticase solution (35 units/ml) and maintained for nine minutes at 30 °C under moderated stirring. The samples were washed three times in PBS and fixed in formaldehyde 3.7% at room temperate for 30 minutes. Then, they were washed three times in PBS and incubated in 10 µM of BJL16#UnB3 (lipid stain molecules) solution for one hour at room temperature. The samples were then washed three times in PBS followed by centrifugation at 3000 g and were resuspended in PBS and seeded in 24 wells plate containing round coverslips pre-treated with 0.1% of Poly-L-lysine in their bottom. The 24 wells plate was centrifuged at 3000 g for 5 minutes and the coverslips were mounted over glass slides by using ProLong® Gold antifade mountant solution (Thermo-Fisher, Waltham, USA). The samples were analyzed in scanning laser confocal microscope, Leica TCS SP5 (New Jersey, USA) under 488 wavelength of laser emission. All assays were performed in triplicate and repeated three times.

#### Cell’s fluorescence analysis

Twenty images from twenty different fields for each sample were randomly acquired. The fields were selected and analyzed based on cells morphologic aspects through bright field image. No fluorescence data was used to select sample images. Three hundred cells were analyzed in triplicate by ImageJ (Maryland, USA) software in order to evaluate the median number of the cells that show fluorescent signal. It was also determined the median number of fluorescent spots for each positive cell to fluorescent signal. The fluorescent spots are equivalent to lipid bodies in cells cytoplasm. It was finally determined the standard deviation to the set of data values. These data were analyzed by GraphPad Prism (San Diego, USA) software in order to evaluate if there are statistically significant differences between samples after nonparametric Mann-Whitney U test (p ≤ 0,01).

#### Fluorescence intensity analysis

The evaluation of fluorescence intensity was performed through generated histogram based on the image lockup table (LUT). The images were also used to create a thermal image, based on image pixels’ values in the LUT. The thermal image was projected as 3D plot over the thermal primary samples images producing a 3D histogram representation over the cellular fluorescent spots, which are the lipids droplets. The fluorescence emission intensity is tightly associated with the lipids amount in lipids droplets. All analyses were performed by using ImageJ software (Maryland, USA).

## Results

### Strains construction

Haploid laboratory *S. cerevisiae* strains CEN.PK 102-3A and CEN.PK 113-6B had their native *SUC2* gene disrupted using KanMX as a selectable marker. As can be seen in Supplementary Fig. S2, primers CH-fwd and CH-rev would generate fragments of approximately 2500 base pairs in strains containing *SUC2* disruption or not, whereas primers CH-fwd and MidK-rev would generate fragments of 1300 base pairs only in strains containing *SUC2* disruption. Successfully disrupted strains were constructed and selected for further manipulations, resulting in strains RH1 and RL1 (Table 2 and Supplementary Fig. 4). In order to evaluate if *SUC2* disruption affected the yeast’s metabolism, the growth rate of strain RH1 was compared to its parental strain’s rate when grown on sucrose. It showed a 15% reduction on the strain’s specific growth rate after disruption.

Strain RH1 was further transformed wither with empty p425GPD plasmid or pGAC (Fig. 2), resulting in intermediate strains RH2 and RH4, respectively (Table 2). In order to assess if the genes in pGAC were being functionally expressed, RmlA activity was measured for RH4 extract samples using RH2 as reference. In cellular extracts of the strains RH2 and RH4, sucrose should be readily converted to glucose 1-Phosphate. Therefore, both sucrose and the substrate of RmlA (glucose-1-phosphate) were used as substrates for the *in vitro* enzyme reaction. When sucrose was used as carbon source, RmlA activity in RH4 extracts was almost 3 times higher than detected on RH2 extracts (Supplementary Fig. S5). However, when glucose-1-phosphate is used as substrate, the two strains showed no significant difference in RmlA activity (Supplementary Fig. S6).

RH and RL strains were further transformed with the remaining plasmids and engineered into a dTDP-L-rhamnose (dTDP-Rha) producing strain (RHP), harboring pGAC and pBD plasmids, and a mono-rhamnolipids (mono-RLs) producing strain (RLP), harboring pGAC, pBD and pAB plasmids. A dTDP-Rha reference strain (RHR) was constructed with the same genetic background as RHP and was transformed with the same plasmids, except they lacked the genes for dTDP-Rha production. Likewise, a mono-RLs reference strain (RLR) was transformed with all three RLP’s plasmids backbones, lacking the genes for mono-RLs production. All yeast strains were deposited in Chalmers Industrial Biotechnology’s yeast strain collection (Fig. 2 for plasmids and Table 2 for strains).

### dTDP-L-rhamnose detection

Strain RHP was tested for dTDP-Rha production using strain RHR as negative control. Strains were grown in YNB medium without aminoacids and 20 g.L^−1^ sucrose, extracted when sucrose was depleted and further analyzed by mass spectrometry. Initially, the experiments of MS and MS/MS were optimized by infusing dTDP-L-rhamnose standard. The best mode of ionization was negative ESI(−)-MS, with the parameters cone voltage, collision energy, and m/z of ion precursor and fragment, configured using MRM mode. The MS triple quadrupole operated in MRM mode makes analysis more sensitive and selective, because it is possible to choose which precursor ion will be fragmented and which fragment will be detected. A peak related to dTDP-Rha was detected in RHP in a MS/MS analysis, while RHR sample showed no such peak (Supplementary Fig. S7). The precursor ion (m/z 547.3) was selected in the first quadrupole (Q1), collided with gas in the second quadrupole (Q2), and the fragment ion of interest (m/z 321) was detected in the Q3. Strains RHR and RHP were analyzed. Extraction was made in triplicate using boiling ethanol method. Then, samples were resuspended in 200 µL water and analyzed by UPLC-MS/MS. In RHP samples, a peak was detected at 3.86 (min) of analysis with MRM transition 547.3 > 321, which can be attributed to dTDP-Rha. The same peak was not detected in the reference RHR strain (red line), strengthening the indication that the compound detected in RHP samples is indeed dTDP-Rha (Supplementary Fig. S7).

### Mono-rhamnolipids detection

**Figure 3:**
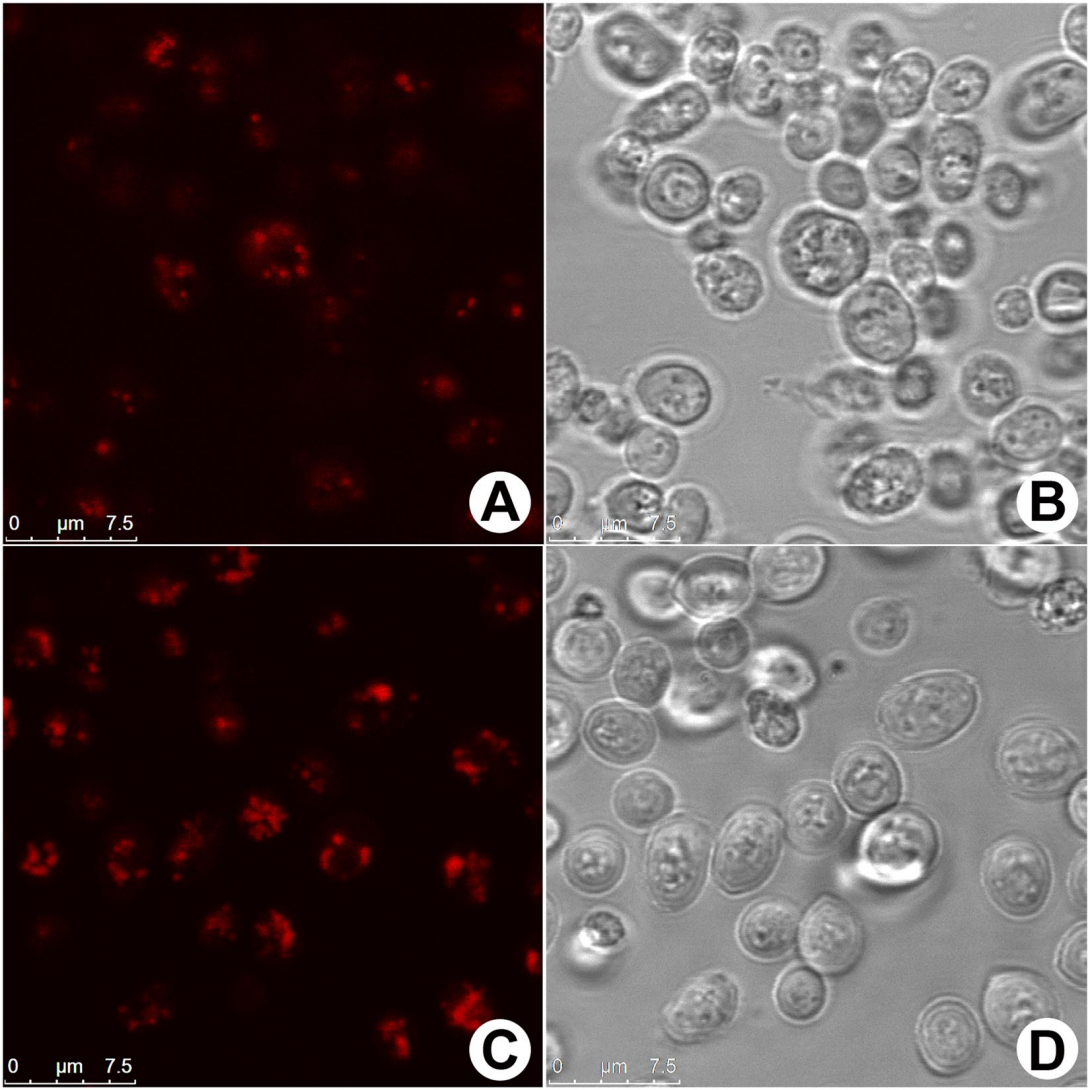
Lipid droplets staining in *Saccharomyces cerevisiae*. The images (**A**) and (**C**) show the lipid droplets accumulated in cells cytoplasm (RLR and RLP strains, respectively). The images (**B**) and (**D**) show the normal morphological aspects of the samples by phase contrast microscopy. Reference scale bar 7.5 μm.

**Figure 4:**
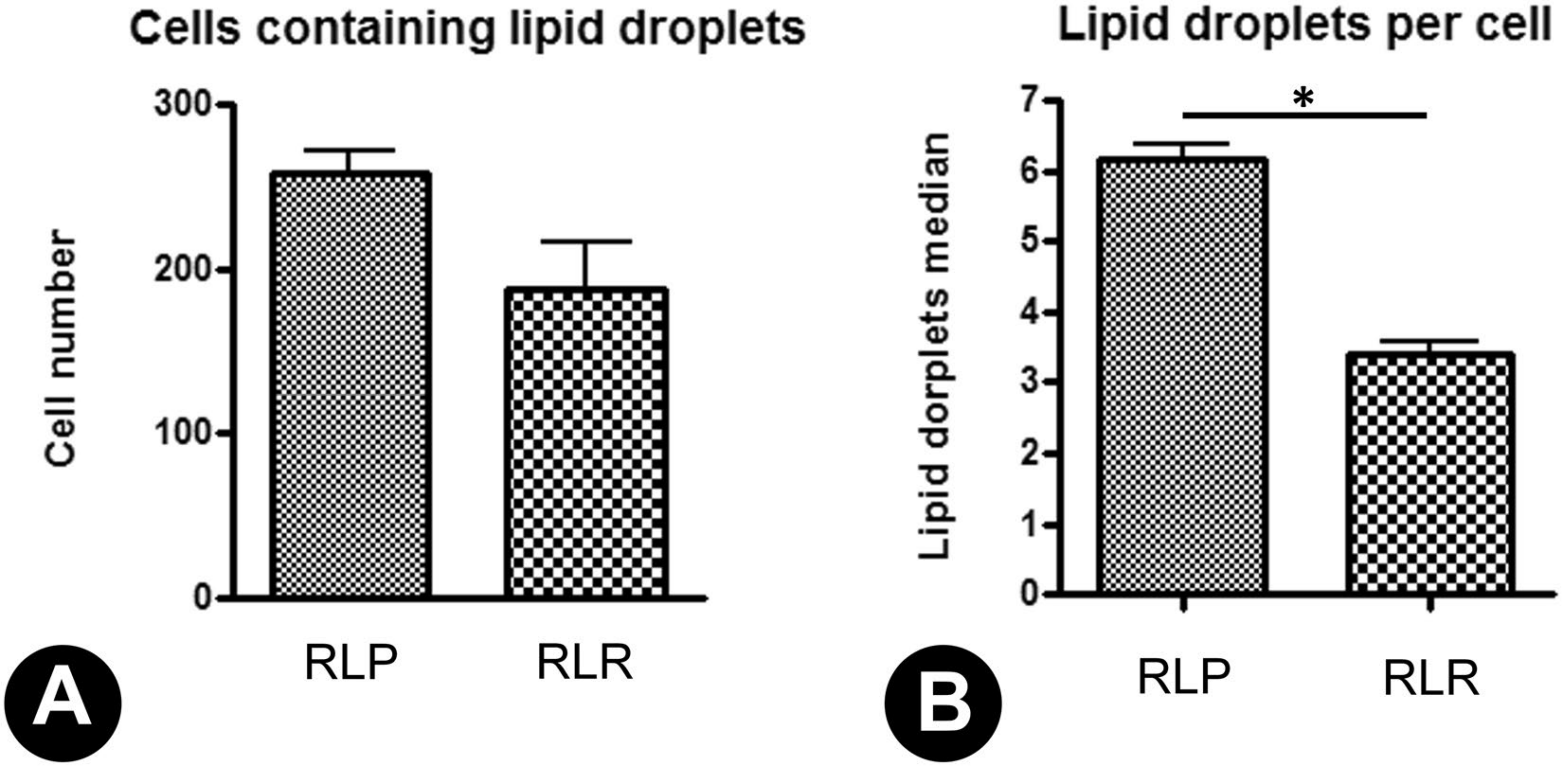
Quantification of lipid droplets production by RLR and RLP strains. The image (**A**) shows the average of cells number containing lipid droplets and the image (**B**) shows the average of lipid droplets found inside the cells. Strains were indicated in x-axis.

Strain RLP was tested for mono-RLs production using strain RLR as negative control. Strains were grown in YNB medium without aminoacids and 20 g.L^−1^ sucrose and, by depletion of sucrose, further analyzed by confocal microscopy. A difference in lipid amounts stored inside the cells in RLR and RLP strains (Fig. 3A and C, respectively) was observed. RLR cells showed apparent reduced number of cells containing lipid bodies and reduced lipid bodies per cell (Fig. 3A) when compared to RLP cells, which showed apparent higher number of lipid bodies per cell and more cells containing lipid bodies (Fig. 3C). Phase contrast images showed the normal morphological aspects of the cells submitted to the staining procedures (Fig. 3B and D). These results were partially confirmed through cellular quantification. Although it seems like there’s an elevated number of RLP cells containing lipid droplets when compared to RLR cells, there’s no statistical difference between the samples due to standard deviation overlapping between strains (Fig. 4A). On the other hand, the number of lipid droplets per cell was approximately two times higher in RLP samples than in RLR samples with statistically significant difference (p < 0,01) (Fig. 4B). Supplementary Figure 7 shows the fluorescent emission profile linked to RLR cells (Supplementary Fig. S8A) and RLP cells (Supplementary Fig. S8B). This profile is tightly associated with the amount of lipids stored inside of each cell. The thermal images (Supplementary Fig. S8C,D) clearly indicated higher lipid accumulation inside lipid bodies in RLP sample. Finally, the histograms (Supplementary Fig. S8E,F) also indirectly indicate higher lipid accumulation in RLP sample compared to RLR sample.

## Discussion

In this study, for the first time, the six genes from *P. aeruginosa* coding for the enzymes responsible for dTDP-Rha and mono-RLs biosynthesis were resynthesized according to codon usage of *S. cerevisiae* and functionally expressed in it, resulting in two final strains: dTDP-Rha producing strain RHP and mono-RLs producing strain RLP. A rhamnose producing strain was constructed because this compound is an important precursor of organic molecules and it is widely used in industrial and laboratorial scale applications, making it an interesting commercial product in addition to RLs. Currently, L-rhamnose production is based on a chemical-dependent synthesis from quercetin, naringin, rutin, polysaccharides and RLs, but these sources are not ideal.^27^.

Initially, the yeast native gene *SUC2* coding for an invertase was disrupted and the *P. saccharophila* native gene Gft coding for an intracellular sucrose phosphorylase was transferred to the yeast, according to a previously reported strategy for free-energy (ATP) conservation^28^. By deleting the invertase and transferring the phosphorylase gene, the yeast no longer hydrolyzes sucrose into glucose and fructose, which would be readily phosphorylated by the yeast, spending an ATP molecule, and directed to the glycolytic pathway. Instead, it internalizes intact sucrose molecules through active transmembrane transport^29^ and converts them into gluc-1-P and fructose using an inorganic phosphate (P_i_), sparing an ATP molecule. The strategy has he additional advantage of yielding directly glucose 1-phosphate, substrate for the first enzyme of the mono-RL’s pathway, RmlA, so the carbon flux doesn’t have to be redirected from glycolysis.

The SUC2 deleted strains were tested for growth on sucrose, showing approximately 10% in specific growth rate reduction, but still able to utilize this carbon source due to the presence of an intracellular maltose invertase. Nevertheless, this doesn’t represent a huge problem since the affinity of sucrose phosphorylase for sucrose (Km 13 mM) is much higher than the one of maltose invertase (Km 116–191 mM)^30,31^, allowing us to speculate that the majority of sucrose would be converted by Gft to glucose 1-phosphate and fructose.

Evaluation of RmlA activity demonstrated a three times higher activity in the RH4 strain than in RH2 when sucrose was used as substrate. However, when dTDP-1-gluc was used for performing the same assay, no significant difference in activity could be detected. It’s important to emphasize that, since RH4 does not have the complete pathway to dTDP-Rha synthesis, dTDP-gluc should accumulate inside the cells due to absence of RmlB. Supplementary Fig. S5 shows the result for the assay performed with sucrose as substrate. As expected, RH4 shows higher dTDP-gluc accumulation, hence higher RmlA activity. On the other hand, Supplementary Fig. S6 shows that, when performed with gluc-1-P as substrate, the assay shows no statistically significant difference for dTDP-gluc accumulation between RH4 and RH2. This can be due to the fact that, when sucrose is used as substrate, besides gluc-1-P, sucrose phosphorylase produces fructose, which can be readily used by glycolysis. When gluc-1-P is used as substrate, it is the only carbon source, so probably it is being redirected to the glycolytic pathway. This can be explained by the presence of the isozymes phosphoglucomutases (PGMs). These isozymes are responsible for the conversion of gluc-1-P to gluc-6-P and vice versa. The major PGM isozyme is PGM2, responsible for 80–90% of PGM activity in yeast, and its high affinity for gluc-1-P was reported as 23.4 μM^32^. It has not yet been reported the affinity of RmlA from *P. aeruginosa*. Nevertheless, it’s reasonable to say that almost all gluc-1-P is being consumed by PGM to feed the glycolytic pathway. Hence, RmlA activity from RH4 fed with gluc-1-P is virtually undetectable.

As it was not possible to determine the other enzymes’ activities in the strains with the complete pathway for dTDP-Rha production, RHP was then directly tested for dTDP-Rha production using RHR as negative control and UPLC-MS/MS. These results show the successful functional expression of the enzymes in the dTDP-Rha pathway. Nevertheless, the amount of compound detected was too little to quantify. This can be due to different factors. As mentioned before, PGM may be competing with RmlA for the gluc-1-P available, decreasing dTDP-Rha yield. Also, as previously reported, dTDP-Rha shows inhibitory effect on RmlA activity when accumulated^33^, decreasing the flux towards dTDP-Rha production. Since dTDP-Rha is not commonly present in yeast’s metabolism, it is not supposed to be redirected to any other pathway, accumulating inside the cells and decreasing cell viability. All these facts show the need to further explore metabolic engineering of RHP, like PGM deletion, to increase dTDP-Rha production or turn it into an L-rhamnose producer by adding extra enzymatic steps to decouple the nucleotide from the rhamnose.

Furthermore, the complete RLs producing strain RLP, which has all 7 heterologously expressed genes, was tested for mono-RLs production by confocal microscopy using RLR as negative control. In this sense, RLP showed no significant difference in the number of cells per sample containing lipid bodies when compared to RLR. However, RLP showed higher number of lipid bodies per cell and higher lipid accumulation inside lipid bodies. These results confirmed that RLP cells are producing and storing more fatty acids than RLR, which is an indication of mono-RLs production and storage. Further specific detection methods need to be explored to quantify RLs production by RLP.

In addition to dTDP-Rha, RLs are synthesized from a beta-hydroxyacyl-ACP. This is an intermediate in the fatty acids *de novo* synthesis pathway already present in yeast cells and can also be a target for metabolic engineering in order to increase RLs production. In this sense, Tang *et al*.^34^ compiled a detailed account of recent advances in metabolic engineering for enhancing fatty acids synthesis in yeast. The main strategies involve enhancing acetyl-CoA and malonyl-CoA production, fatty acids precursors; inhibit or decrease the flow in glycerol and ethanol pathways, which compete for the carbon source; decrease in-pathway negative feedback; block beta-oxidation, which consumes fatty acids; enhancing intracellular concentrations of NADPH, necessary for fatty acids synthesis. As can be seen, there are many advances in this topic, which can be applied to the strains constructed in this study to further improve RLs production.

Moreover the development of a yeast-based process for the RLs production could be further integrated in the biorefinery concept largely utilized in Brazil. In this regard, the strains here presented could be further developed to utilize C5 sugars for RLs production from cellulosic residues. GranBio is a Brazilian biotech company, which was the first to produce second generation (2 G) bioethanol in the Southern Hemisphere, with an industrial plant capable of producing 82 million liters per year^35^. As of 2015, GranBio received approval for using its own proprietary yeast capable of fermenting C5 and C6 sugars^36^. Coupling metabolic engineering strategies to produce other molecules beside 2 G ethanol can further decrease the carbon footprint and add value to the vast biomass residues available in the country.

In conclusion, this study shows, for the first time as a proof of concept, that *Saccharomyces cerevisiae* can be engineered into a rhamnose or rhamnolipids producer. Current efforts are focused on trying to characterize and quantify the products in the strains in order to keep further improving the production with new approaches. The strategy here presented is an important development towards improving industrial production of rhamnolipids using an inexpensive substrate and a GRAS eukaryotic host.

## Electronic supplementary material

Supplementary Information
