# Supplementary material for: Rhamnolipids production from sucrose by engineered Saccharomyces cerevisiae
Source: Sci Rep. 2018 Feb 13;8:2905. doi: 10.1038/s41598-018-21230-2 (PMC5811566; doi:10.1038/s41598-018-21230-2)

Rhamnolipids production from sucrose by engineered *Saccharomyces cerevisiae*

Frederico Mendonça Bahia Silva[1,+], Gabriela Almeida[2,+], Lorena Pereira de Andrade[1], Christiane Gonçalves Campos[3,4], Lúcio Rezende Queiroz[1], Rayane Luzia Vieira da Silva[2], Patrícia Verardi Abdelnur[3,4], José R Corrêa[1], Maurizio Bettiga[5,6], and Nádia Skorupa Parachin[1,+,*]

[1] Department of Molecular Biology, Biological Sciences Institute, University of Brasília (UnB), Campus Darcy Ribeiro, Block K. Postal code: 70.790-900. Brasilia, Federal District, Brazil.

[2] Catholic University of Brasília (UCB), Advanced Campus Asa Norte, SGAN 916 Block B Avenue W5. Postal code: 70.790-160. Brasilia, Federal District, Brazil.

[3] Brazilian Agricultural Research Corporation, Embrapa Agroenergy, W3 Norte, PqEB, Postal code: 70770-901, Brasília, Federal District, Brazil.

[4] Institute of Chemistry, Federal University of Goiás, Campus Samambaia, Postal code: 74690-900, Goiânia, Goiás, Brazil.

[5] Department of Biology and Biological Engineering, Division of Industrial Biotechnology, Chalmers University of Technology. SE-41296, Gothenburg, Sweden.

[6] EviKrets Biobased Processes Consultants, Gibraltarsgatan 40, 41280 Gothenburg, Sweden

[*] nadiasp@gmail.com

[+] these authors contributed equally to this work

Supplementary Information

Supplementary Table S1. **Title.** Primers used in the study. Primers listed as _cassette were used in amplifying cassettes fragments for plasmid construction. Primers listed as _confirm were used for plasmid construction validation.

Name	Sequence	Tm (°C)
rmlAF_cassette	AATGCGGCCGCAAATTAAAGCCTTCGAG	65.2
rmlAR_cassette	ATTGCGGCCGCAAAATGTTTCTACTCCTT	62.7
rmlCF_cassette	GTAGAGCTCGCTCATTTGGCGAGCGTTG	65.8
rmlCR_cassette	GTAGAGCTCGGGCGAATTGGGTACCGGC	67.3
rmlDF_cassette	GTAGAGCTCGTACCGGCCGCAAATTAAA	62.4
rmlDR_cassette	GTAGAGCTCCATAGCTTCAAAATGTTTCT	59.7
rhlBF_cassette	ATAGGTACCGCGCAATTAACCCTCAC	52.9
rhlBR_cassette	TATAGGCGAATTGGGTACC	52.7
GftF_confirm	TCACATACGTAGATAGGTTGG	51.9
GftR_confirm	GAGAATGCAAAGGCTCTATC	52.2
rmlAF_confirm	GAGGAAAGGTATCATATTGGC	51.9
rmlAR_confirm	AGTAGACAGTTTCAGTCAACA	51.5
rmlBF_confirm	GACTATATTGGTTACAGGTTCAG	51.3
rmlBR_confirm	CATGCATACTGTTTACCTACC	51.5
rmlCF_confirm	AGCTACTAGATTGGCTATCC	51.9
rmlCR_ confirm	TTATGGGAAACAATCAGCATC	51.7
rmlDF_ confirm	GAATCTTATTATTGGGAGCCAA	51.4
rmlDR_ confirm	TCACAATGGTCCCTGTT	51.4
rhlAF_confirm	ATGAGAAGGGAAAGTTTATTGG	51.2
rhlAR_confirm	GGCATAGCCGATAGC	51.1
rhlBF_confirm	GCACGCCATCTTGATAG	51.8
rhlBR_confirm	TTAAGAAGCAGCCTTCAAC	51.4

Supplementary Figure S2. **Title.** SUC2 gene disruption strategy. **Legend.** Small arrows indicate primers. In red, disruption primers. In green, verification primers, which anneal 500 base pairs upstream and downstream from *SUC2* locus in yeast's Chromosome IX. In blue, verification primer, which anneal inside KanMX cassette, 1300 base pairs away from CH-fwd annealing site.

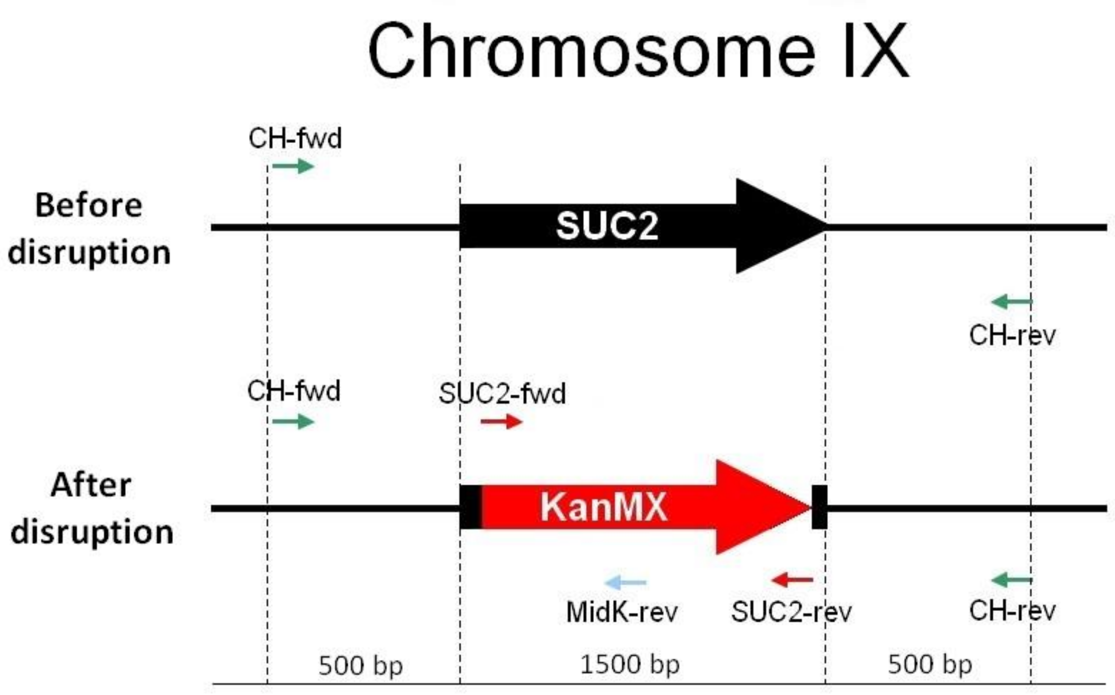

	Name	Sequence
**Disruption primers**	SUC2-fwd	TCTCTCAGAGAAACAAGCAAAACAAAAAGCTTT TCTTTTCCAGCTGAAGCTTCGTACGC
	SUC2-rev	GCTAAAGCCCTTTAGAATGGCTTTTGAAAAAAA TAAAAAGCATAGGCCACTAGTGGATCTG
**Verification primers**	CH-fwd	GTTTAGGAAATTATCCGGGGGC
	CH-rev	GCTTGGTATCCATTTCCCTCAC
	MidK-rev	CGCGATCGCTGTTAAAAGGAC

Supplementary Table S3. **Title.** Bacterial strains used and constructed in this study: name, relevant genotype and parental strain.

Name	Relevant genotype	Parental strain
DH5-α	-	-
Ec-G	pBSK Gft	DH5-α
Ec-A	pBSK RmlA	DH5-α
Ec-B	pBSK RmlB	DH5-α
Ec-C	pBSK RmlC	DH5-α
Ec-D	pBSK RmlD	DH5-α
Ec-HA	pBSK RhlA	DH5-α
Ec-HB	pBSK RhlB	DH5-α
Ec-CasG	p425GPD Gft	DH5-α
Ec-CasA	p426TEF RmlA	DH5-α
Ec-CasB	p426GPD RmlB	DH5-α
Ec-CasC	p426CYC1 RmlC	DH5-α
Ec-CasD	p416TEF RmlD	DH5-α
Ec-CasHA	p424TEF RhlA	DH5-α
Ec-CasHB	p424ADH RhlB	DH5-α
Ec-CasGA	p425GPD Gft RmlA	DH5-α
Ec-CasGAC	p425GPD Gft RmlA RmlC	DH5-α
Ec-CasBD	p426GPD RmlB RmlD	DH5-α
Ec-CasHAB	p424TEF RhlA RhlB	DH5-α

Supplementary Figure S4. **Title.** *SUC2* gene disruption confirmation. **Legend.** Electrophoresis from colony PCRs confirming *SUC2* disruption in CEN.PK 102-3A (A) and CEN.PK 113-6B (B) strains. M: 1kb plus marker from ThermoScientific; C-: Water; C+: Parental strain; An: colony samples picked after parental strains transformations with KanMX and selection in YPD plates containing geneticin.

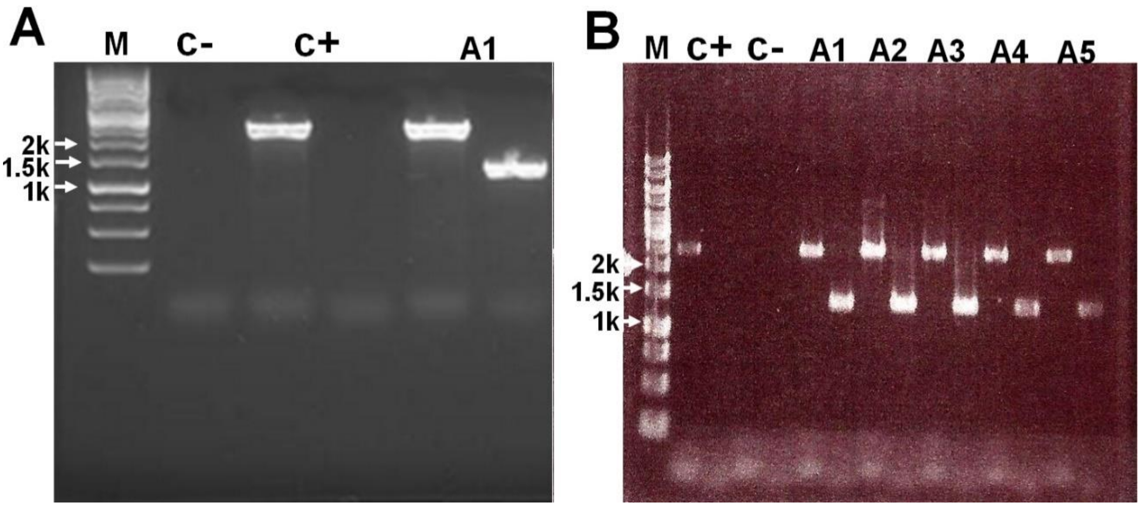

Supplementary Figure S5. **Title.** RmlA activities in RH2 and RH4 strains with sucrose as substrate. **Legend.** Activity detected in RH4 strain is significantly higher than in RH2 strain.

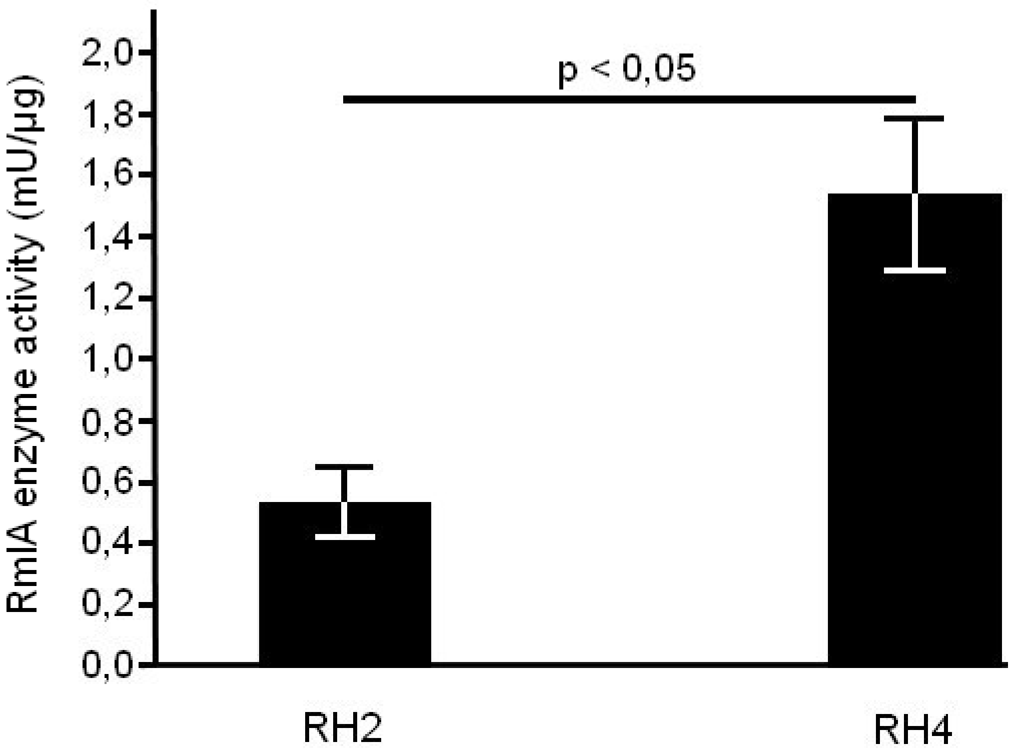

Supplementary Figure S6. **Title.** RmlA activities in RH2 and RH4 strains with glucose-1-phosphate as substrate. **Legend.** Activities are not statistically different between the strains.

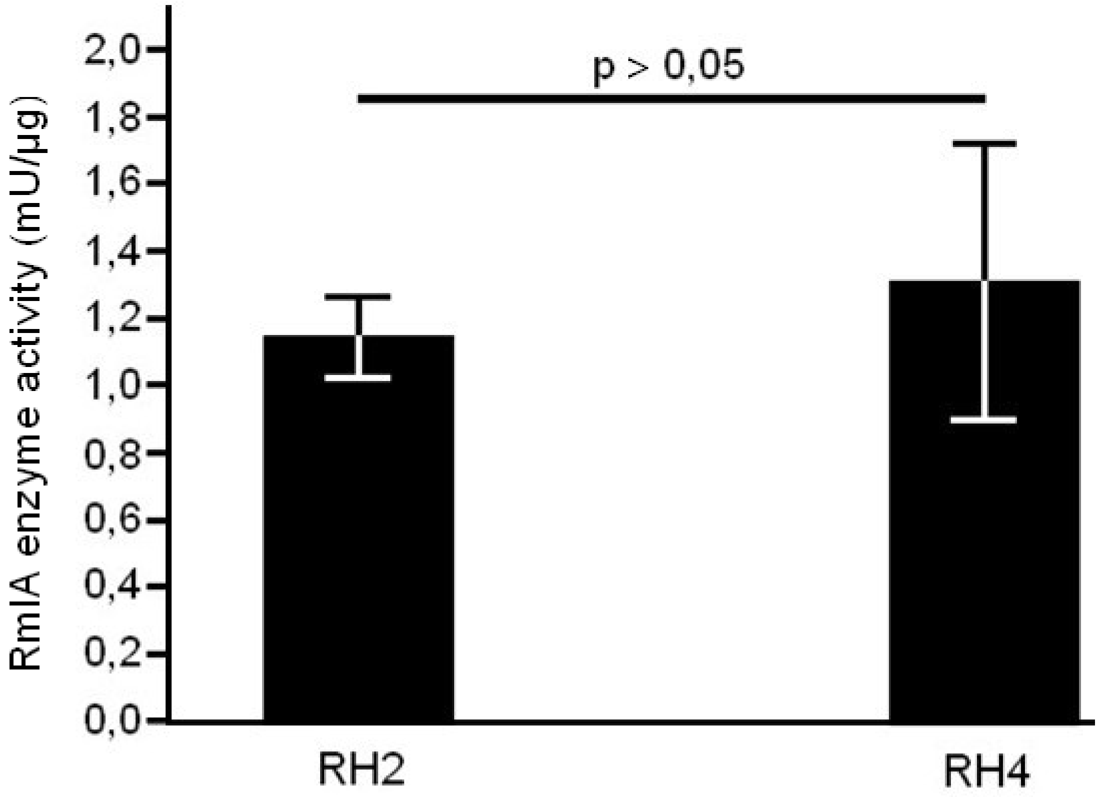

Supplementary Figure S7. **Title.** MRM results for RHR and RHP strains. **Description.** In RHP samples (black line), dTDP-Rha (547.3 > 321) is eluted after 3.86 minutes of chromatographic analysis. RHR samples (red line) show no such elution peak.

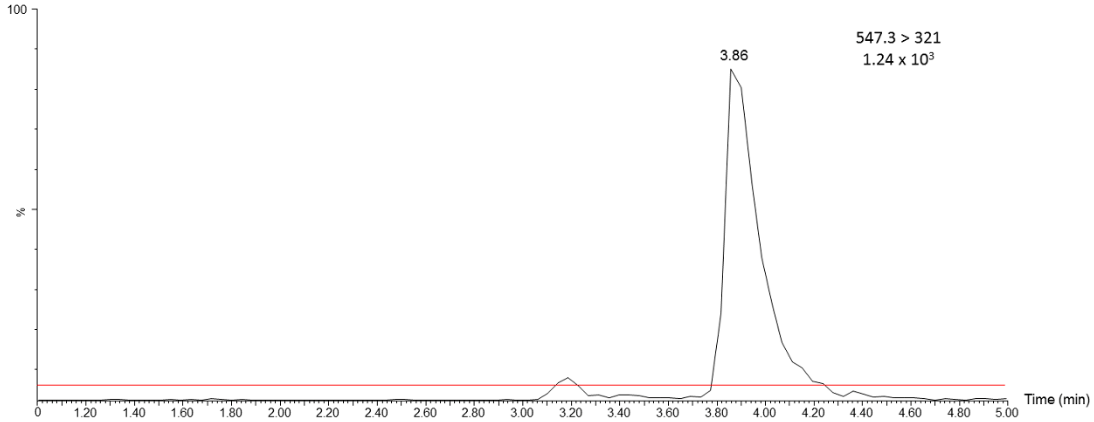

Supplementary Figure S8. **Title.** Fluorescence signal quantification. **Legend.** Images A and B show the fluorescent profile of RLR and RLP cells, respectively. Images C and D show the 3D plot of thermal representation of pixel values obtained from the images LUT. Images E and F show the histogram related to pixel value obtained from images LUT.

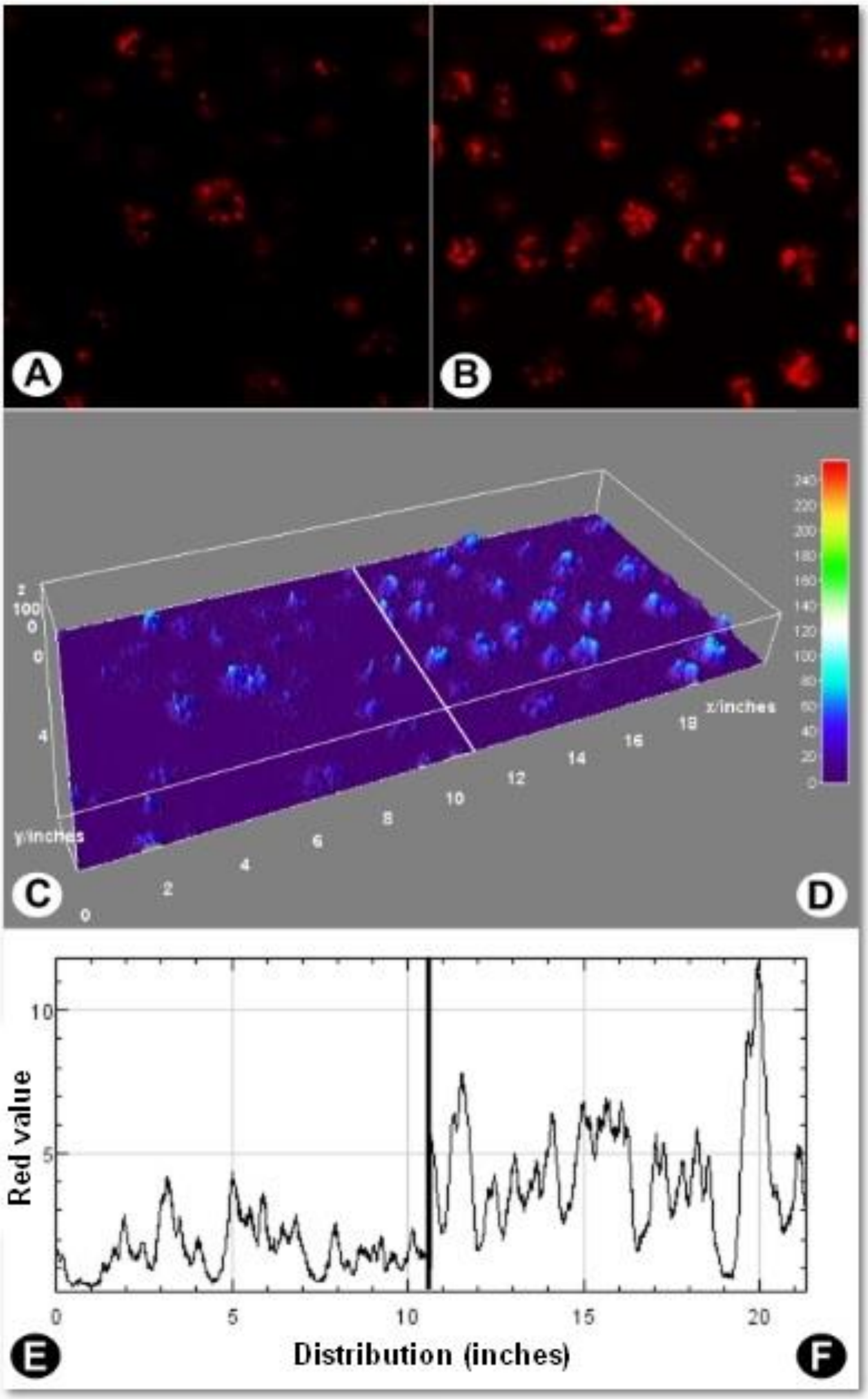

Supplement: Supplementary file 1 — Supplementary Information [file 41598_2018_21230_MOESM1_ESM.pdf]
